# Efficient Differentiation of Steroidogenic and Germ-Like Cells from Epigenetically-Related iPSCs Derived from Ovarian Granulosa Cells

**DOI:** 10.1371/journal.pone.0119275

**Published:** 2015-03-09

**Authors:** Raymond Anchan, Behzad Gerami-Naini, Jennifer S. Lindsey, Joshua W. K. Ho, Adam Kiezun, Shane Lipskind, Nicholas Ng, Joseph A. LiCausi, Chloe S. Kim, Paul Brezina, Thomas Tuschl, Richard Maas, William G. Kearns, Zev Williams

**Affiliations:** 1 Division of Reproductive Endocrinology and Infertility, Department of Obstetrics, Gynecology and Reproductive Biology, Brigham and Women’s Hospital, Harvard Medical School, Boston, Massachusetts, United States of America; 2 Division of Genetics, Department of Medicine, Brigham and Women’s Hospital, Harvard Medical School, Boston, Massachusetts, United States of America; 3 Victor Chang Cardiac Research Institute and the University of New South Wales, Sydney, New South Wales, Australia; 4 Computational Methods Development, Cancer Genome Analysis, Broad Institute of MIT and Harvard, Cambridge, Massachusetts, United States of America; 5 Department of Gynecology and Obstetrics, John’s Hopkins University School of Medicine, Baltimore, Maryland, United States of America, and Center for Preimplantation Genetics, LabCorp, Rockville, Maryland, United States of America; 6 Laboratory of RNA Molecular Biology, Howard Hughes Medical Institute, The Rockefeller University, New York, New York, United States of America; 7 Division of Reproductive Endocrinology and Infertility, Department of Obstetrics, and Gynecology and Women’s Health, Albert Einstein College of Medicine, Bronx, New York, United States of America; Northern Institute for Cancer Research, UNITED KINGDOM

## Abstract

To explore restoration of ovarian function using epigenetically-related, induced pluripotent stem cells (iPSCs), we functionally evaluated the epigenetic memory of novel iPSC lines, derived from mouse and human ovarian granulosa cells (GCs) using *c-Myc*, *Klf4*, *Sox2* and *Oct4* retroviral vectors. The stem cell identity of the mouse and human GC-derived iPSCs (mGriPSCs, hGriPSCs) was verified by demonstrating embryonic stem cell (ESC) antigen expression using immunocytochemistry and RT-PCR analysis, as well as formation of embryoid bodies (EBs) and teratomas that are capable of differentiating into cells from all three germ layers. GriPSCs’ gene expression profiles associate more closely with those of ESCs than of the originating GCs as demonstrated by genome-wide analysis of mRNA and microRNA. A comparative analysis of EBs generated from three different mouse cell lines (mGriPSCs; fibroblast-derived iPSC, mFiPSCs; G4 embryonic stem cells, G4 mESCs) revealed that differentiated mGriPSC-EBs synthesize 10-fold more estradiol (E2) than either differentiated FiPSC- or mESC-EBs under identical culture conditions. By contrast, mESC-EBs primarily synthesize progesterone (P4) and FiPSC-EBs produce neither E2 nor P4. Differentiated mGriPSC-EBs also express ovarian markers (AMHR, FSHR, Cyp19a1, ER and Inha) as well as markers of early gametogenesis (Mvh, Dazl, Gdf9, Boule and Zp1) more frequently than EBs of the other cell lines. These results provide evidence of preferential homotypic differentiation of mGriPSCs into ovarian cell types. Collectively, our data support the hypothesis that generating iPSCs from the desired tissue type may prove advantageous due to the iPSCs’ epigenetic memory.

## Introduction

Embryonic stem cells (ESCs) hold great promise for therapeutic and regenerative medicine applications due to their inherent ability to produce tissue from all three germ layers. However, ESCs can only be produced from discarded human embryos generated during fertility treatment. More recently, the emergence of protocols that derive induced pluripotent stem cells (iPSCs) from somatic tissue has revolutionized stem cell research by affording alternatives to embryo-derived ESCs [1, 2]. With this discovery, we now have an alternate population of pluripotent stem cells that may be derived from a variety of terminally differentiated somatic cells.

The ability to generate stem cells from adult tissue offers hope to patients by facilitating autologous stem cell therapies [3, 4]. Yet a significant scientific hurdle to using ESCs or iPSCs in regenerative medicine is the paucity of information on the precise molecular signals necessary to direct differentiation into specific tissues. Despite their overall similarity to ESCs, accumulating evidence suggests that iPSC lines vary in their capacity to produce certain tissue types upon spontaneous differentiation [5–8]. This restriction is in part related to reprogrammed cells’ epigenetic signature, which may be defined as a mechanism by which cells retain a functional memory of their originating identity throughout cell divisions [9–12]. While one metric of such memory involves methylation analysis of tissue-specific genes, here we demonstrate a functional analysis of differentiating stem cells. With these techniques we test our hypothesis that tissue-specific iPSCs favor homotypic differentiation, reverting preferentially to their originating cell type.

The consequence of this epigenetic memory has been noted in the biased spontaneous differentiation of iPSCs towards their originating tissue type [13, 14]. For example, when blood-derived iPSCs are allowed to spontaneously differentiate, they are four times more likely to revert to a hematopoietic phenotype than non-blood-derived iPSCs [15]. While this may restrict the utility of blood-derived iPSCs to readily produce non-hematopoietic cell types, such as neural or endodermal cells, their epigenetically-influenced differentiation is advantageous when generation of blood cells is the goal. Thus derivation of tissue-specific iPSCs for homotypic differentiation may be beneficial for targeted regenerative therapies.

In the context of reproductive medicine, these novel techniques employing iPSCs could be used to restore ovarian function in women with premature ovarian failure (POF). POF, a condition characterized by loss of ovarian function before age 40, has been associated with a number of genetic and environmental influences, including Fragile X premutations, 45X/46XX low level mosaicism, and autoimmune and infectious oophoritis. Iatrogenic ovarian injury due to adnexal surgery, pelvic radiation, or chemotherapy is also a major concern. For example, 1 in 8 women in North America are diagnosed with breast cancer and nearly 30% of these women are of reproductive age. In these patients who are under age 30, 10% will suffer complete ovarian failure post-chemotherapy due to the gonadotoxic effects of the chemotherapeutic agents, and 100% of women over age 40 will have long-term, if not permanent, chemotherapy-related amenorrhea [16–19]. In order to investigate the potential of stem cell-based ovarian regeneration therapies, we utilize ovarian granulosa cells (GCs) to generate iPSCs for purpose of differentiating functional endocrine tissue and assess for evidence of preferential epigenetic memory (S1 Fig.).

GCs are one of the three distinct, functional cell types that comprise the mammalian ovary, along with thecal cells and primordial oocytes. These cell populations are arranged in a specific configuration to form ovarian follicles, the sites of steroidogenesis and oocyte maturation in the adult ovary. The juxtaposition of GCs next to the developing oocyte reflects the functional interdependence of these cell types. GCs support oocyte maturation and secrete estrogens while the oocyte in turn secretes signals to regulate GCs [20–24]. During assisted reproductive technologies (ART), such as *in vitro* fertilization (IVF), oocytes are retrieved in the form of an oocyte-GC-complex. GCs are typically isolated and discarded when oocytes are fertilized to generate the embryos used in the patients’ treatment. Per the Society for Assisted Reproductive Technology Clinical Outcome Reporting System (SART-CORS) report, during 2011 there were over 140,000 IVF cycles performed in the United States [25]. Thus discarded GCs are an abundant and accessible source of somatic cells that may be reprogrammed into iPSCs and used in regenerative therapies, especially to address health issues related to ovarian function.

In this study, we demonstrate that GCs of the mouse and human ovary are readily reprogrammed into iPSCs. Both mouse and human GC-derived iPSCs (mGriPSCs; hGriPSCs) share antigenic markers of embryonic stem cells, exhibit pluripotent differentiation capability, and spontaneously form embryoid bodies (EBs). These cells also demonstrate preferential homotypic differentiation into ovarian cell types, supporting the hypothesis that the epigenetic memory of tissue-specific iPSCs may limit their pluripotency and differentiation potential. Here, we show that the mGriPSCs generate steroidogenic cells akin to ovarian endocrine cells, as previously reported for mouse and human ESCs [26, 27]. Moreover, the mGriPSCs can generate cells expressing ovarian and primordial germ cell markers at a greater efficiency than that observed in mESCs.

## Materials and Methods

All supplies were purchased from Sigma-Aldrich (St. Louis, MO) and all antibodies were purchased from Abcam (Cambridge, MA; S1 Table), unless otherwise noted.

### Cell lines and animals

G4 mouse Embryonic stem cells (mESC) were pruchased from the Lunenfeld-Tanenbaum Research Institute [28], mouse fibroblast induced pluripotent stem cell line (FiPSC) was obtained from System Biosciences [29] and mouse granulosa cell derived-iPSCs were generated in our laboratory. All female C57BL6/J mice were purchased from Charles River Laboratories (Wilmington, MA).

### Collection of granulosa cells

#### Mouse granulosa cells collection

With IUCAC approval, pregnant mare’s serum gonadotropin [PMSG]-hyperstimulated female C57BL6/J mice were sacrificed and oocyte-GC-complexes were harvested from the fallopian tubes using standard techniques. Cumulus GCs surrounding each oocyte were released using hyaluronidase and collected (approximately 200,000 cells per collection). GCs were centrifuged at 1500 rpm to pellet and cultured on gelatin-coated plates in defined GC media containing DMEM-F12, 50 μg/ml sodium pyruvate (GIBCO, Carlsbad, California), 5 ng/ml insulin, 0.01 IU/ml PMSG, 5 μg/ml transferrin, 10μg/ml selenium, and 10% heat-inactivated fetal bovine serum (HI FBS, Hyclone, South Logan, UT).

#### Discarded human granulosa cells collection

De-identified, discarded cumulus granulosa cells were obtained during egg-retrievals from consenting patients undergoing routine fertility treatment procedures in the *in vitro* fertilization clinical laboratory at Brigham and Women’s hospital (BWH). GCs were separated from the oocytes using hyaluronidase as per standard clinical treatment protocols and cultured in GC media (between 1–3 million GCs per collection).

### Generation of retrovirus vectors and iPSCs

Mouse and human retroviral reprogramming vectors for the iPSC genes *Oct4*, *Sox2*, *c-Myc* or *Klf4* were generated using standard retroviral production protocols using pMXs retroviral plasmids [30] (Addgene, Cambridge, MA). 293T cells were cultured in DMEM and 10% HI FBS until 40–50% confluent, and then transfected with the reprogramming vectors and VSV-G using FuGENE (Roche, Indianapolis, IN) for 48 hours. Virion particles were then harvested. The mouse and human retroviral vectors were generated using ecotropic and amphotropic virus, respectively. Fresh viral vectors are then employed to infect target GCs primary cultures along with the 6 μg/ml polybrene (Millipore, Billerica, MA). Mouse GCs tend to not proliferate well in culture, so primary cells were infected. Using the Yamanaka retroviral technique, which is replication-dependent, resulted in low infection efficiency. Efficiency was comparable to other published attempts at mouse iPSC generation, at 0.001–0.01% [2, 31, 32]. This variability in derivation efficiency was largely related to the poor *in vitro* proliferation of GC cells prior to reprogramming. After 24 hours, the viral media was rinsed and cultures observed for 2 weeks for emergence of stem cell-like colonies. Putative iPSC colonies were manually picked based on morphology and subcultured on feeder layers of mouse embryonic fibroblasts [MEFs] cells [Global Stem, Rockville, MD] that are mitotically-inactivated with mitomycin C. All stem cells cultures were grown in standard mouse or human stem cell media. Mouse ES media was comprised of DMEM, 10% ES-grade HI FBS, 1000 U/ml ESCRO LIF (Millipore), 2 mM L-Glutamine [GIBCO], 0.2 mM 2-mercaptoethanol. Human ES media consisted of DMEM-F12, 20% Knock Out Serum Replacement (KOSR, GIBCO), 1 mM L-Glutamine, 0.1 mM 2-mercaptoethanol, 8 ng/ml bFGF (Invitrogen, Grand Island, NY).

### mGriPSC and hGriPSC colony expansion

**Fig 1:**
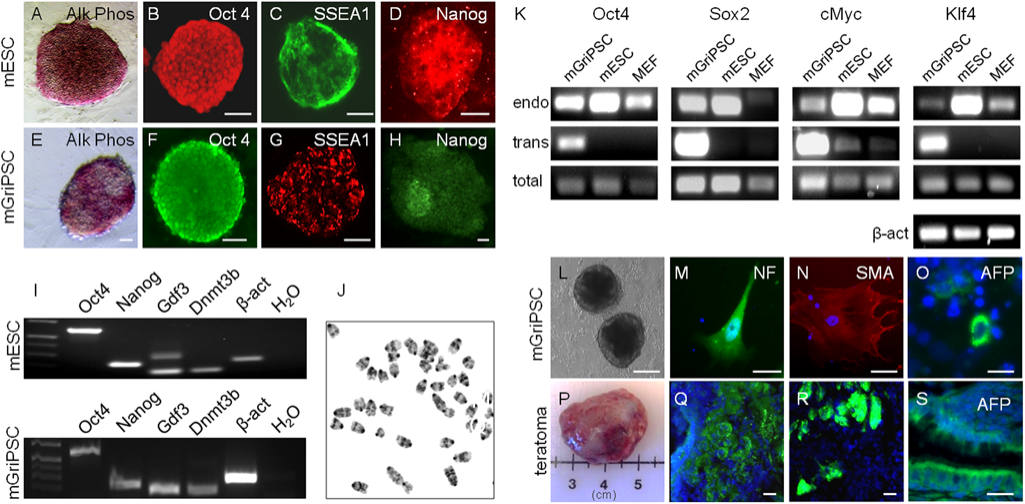
mGriPSCs are pluripotent. Using G4-mESCs as a standard **(A-D, I)**, mGriPSCs are alkaline phosphatase reactive **(E)** and express stem cell antigens Oct4 **(F)**, SSEA1 **(G)** and Nanog **(H, I)**. mGriPSCS express additional stem cell markers by RT-PCR **(I)** and are karyotypically normal **(J)**. mGriPSC express endogenous copies of the introduced reprogramming genes and retroviral [trans] copies are present to varying degrees **(K).** mGriPSCs form EBs **(L)** that differentiate into three germ layers—ectodermal neurofilament **(M)**, and mesodermal SMA **(N)**, and endoderm alpha-fetoprotein (**O,** AFP). Teratomas **(P)** also show differentiation into three germ layers **(Q-S)**. Scale bars: **A-H** 20 μm; **L** 200 μm; **M-O, Q-S** 10 μm.

Primitive colonies of iPSCs were grown on MEFs for several days and manually picked based on stem cell colony morphology and passaged onto fresh feeder plates. Further subculture was based on live-immunostaining screening [33], in which live colonies were immunostained while in culture for an external antigen that is characteristic of undifferentiated stem cells. Mouse embryonic stem cell marker stage-specific embryonic antigen-1 (SSEA-1, Millipore) was used for mGriPSC colonies (Fig. 1E-H) and human embryonic stem cell markers SSEA-4 and TRA-1–60 (Millipore) were used for hGriPSC colonies (S2 Fig.). Primary antibody was used at a 1:100 dilution in stem cell media at 37°C overnight followed by incubation in a 1:1000 dilution of secondary rhodamine- or fluoroscein-tagged antibodies in fresh stem cell media for a minimum of 2 hours at 37°C. Positively immunolabeled live colonies were then rinsed of antibody and expanded for further characterization.

### Verification of mGriPSC and hGriPSC stem cell status

Cultures of manually selected, presumptive mouse and human GriPSC colonies were fixed using 4% paraformaldehyde (4°C)/4% sucrose in 0.1 N PBS for 15-minutes at room temperature and rinsed twice with 0.1N PBS for 5-minutes. An alkaline phosphatase reaction kit [Millipore] was used to evaluate enzyme activity in the iPSC colonies. Stem cell status was also assessed by immunocytochemistry (ICC) using commercial stem cell antibodies for mouse Oct4, SSEA-1, and Nanog, and for human OCT4, SSEA-4, TRA-1–60 and TRA-1–81 (Millipore; S2 Fig.; S1 Table). Differentiation was analyzed by ICC using antibodies to neurofilament [NF, ectoderm], alpha fetoprotein (AFP, endoderm; Santa Cruz, Santa Cruz, CA), and alpha smooth muscle actin (SMA, mesoderm; S1 Table). Controls included immunostaining for stem cell antigens on primary GCs to assess for expression of these genes prior to infection (S3 Fig.). Additional controls included omission of primary or secondary antibodies.

RNA was extracted using commercially available kits (Qiagen, Germantown, MD). Reverse transcription of cDNA was performed using qScript cDNA Synthesis (Quanta Biosciences, Gaithersburg, MD) and PCR was performed to confirm expression of additional stem cell markers. In the mGriPSCs, expression of Oct4, Nanog, SSEA1, Gdf3, and Dnmt3b were investigated using GoTaq Flexi polymerase (Promega, Madison, WI). For the hGriPSC line, the expression of OCT4, NANOG, DNMT3B, and GDF9 (S2 Table) were tested.

Absence of chromosomal abnormalities was determined by the Dana Farber/Harvard Cancer Center Cytogenetics Core facility (Boston, MA) in ten metaphase cells from mGriPSC colonies (Fig. 1J).

This process generated a total of 12 positively identified and characterized mGriPSC clones.

### PCR analysis of reprogrammed iPSC line

**Fig 2:**
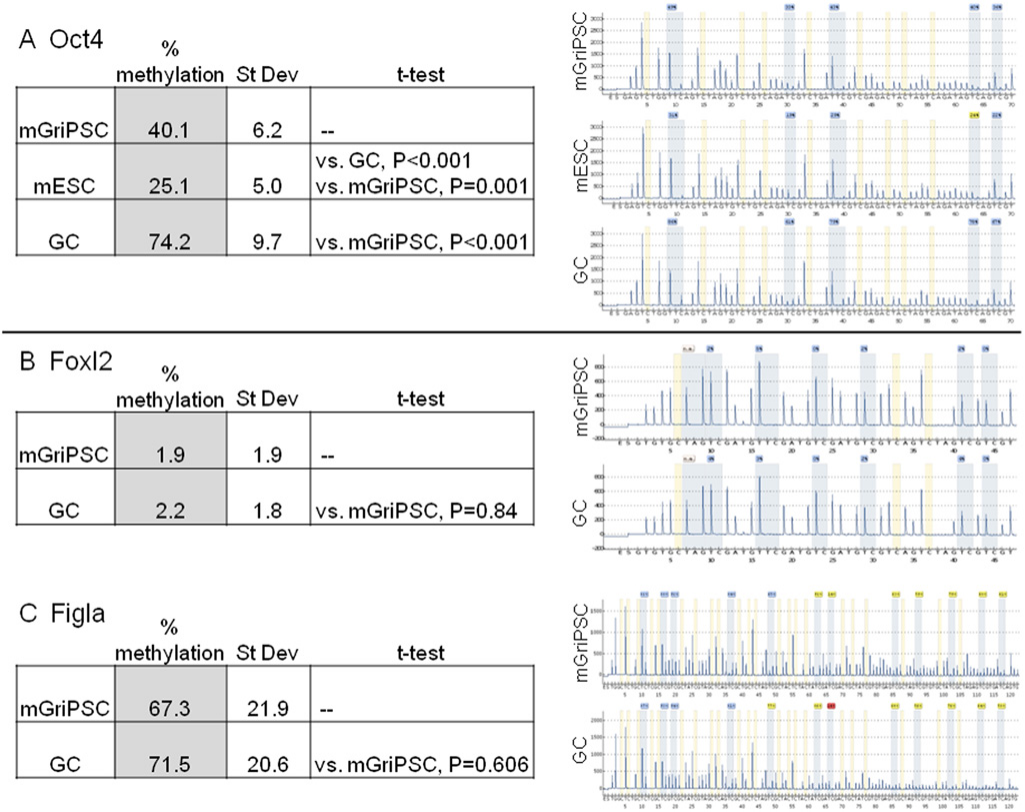
Methylation analysis of GC-derived iPS cells shows stem cell reprogramming with retention of target tissue methylation. The stem cell gene Oct4 in mGriPSC is less methylated than it is in GCs (mGriPSC vs. GC, P<0.001) and is therefore more similar to Oct4 in G4 ESCs **(A)**. Methylation patterns of ovarian and germ cell genes Foxl2 and Figla are comparable in both mGriPSC and GCs (**B**, Foxl2, P = 0.84; Figla, P = 0.606).

Endogenous (native cellular), transgene (reprogramming vector), and total (capturing both) primers for all four stem cell genes that were introduced to the cells were used to determine the origin of stem cell gene expression (Fig. 2; S2 Table). These primers were used to characterize our mGriPSC line, G4 mESCs and control MEF cells. RT-PCR was performed using the kits and protocols described above, with the addition of 5% DMSO to the PCR reactions.

### Teratoma formation

Low-passage mGriPSCs cultured on irradiated MEFs [Global Stem] were detached using with collagenase IV (GIBCO) (1 mg/ml collagenase IV dissolved in 37°C DMEM media, filtered through 0.22 micron syringe filter) until detachment of the edges of the mGriPS colonies was detected. These colonies were harvested and dissociated with Accutase (Invitrogen) for 2–3 minutes to produce a single-cell suspension. Approximately 5–9 million mGriPSC cells in 100 μl DMEM media (pre-warmed at 37°C) were injected into the rear leg muscles of five-week-old severe combined immunodeficient (SCID) mice. A total of six mice were injected. Mice were sacrificed 7–8 weeks after injection and teratoma tissue was excised, fixed in cold 4% paraformaldehyde/4% sucrose, and processed for paraffin embedding. Sectioned slides containing various regions of tumors were stained by hematoxylin and eosin (H&E). Complex structures with various cell types were analyzed. Teratoma sections were also immunostained for differentiation markers (listed above).

This study was carried out in strict accordance with the recommendations in the Guide for the Care and Use of Laboratory Animals of the National Institutes of Health. The protocol was approved by the Harvard Medical Area Standing Committee on Animals (Protocol: 750). All efforts were made to minimize suffering.

### Embryoid body formation and endocrine assays

**Fig 3:**
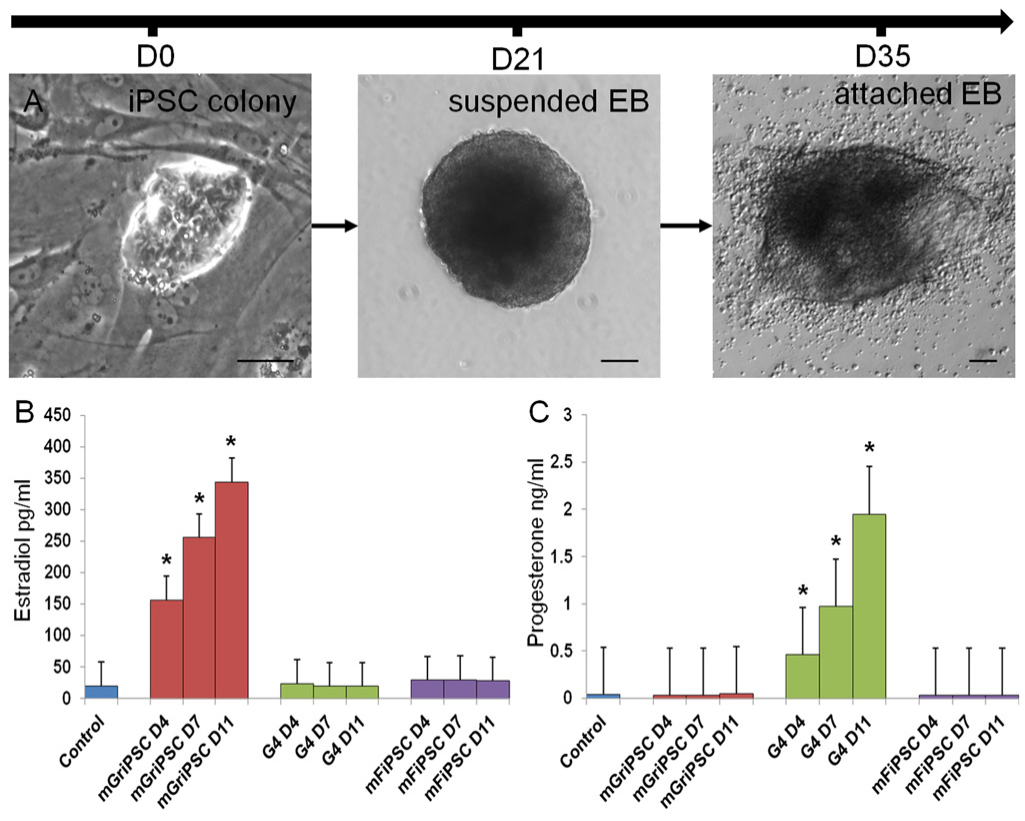
mGriPSC-EBs preferentially synthesize estradiol. Mouse GCs **(A)** infected with pluripotency genes generate iPSC colonies. Arrow indicates primary granulosa cells. mGriPSC-EBs, when allowed to attach, synthesize estradiol **(B)** at concentrations 5 to 12 times those of G4-EBs and mFiPSC-EBs. Two-tailed t-test, *P<0.001, different from other two cell lines at same time point. G4-EBs primarily secreted progesterone **(C)** while mFiPSC-EBs produced negligible amounts of either. Two-tailed t-test, *P<0.005, different from other two cell lines at same time point. Scale bar: **A** 100 μm.

EBs were generated from mGriPSC (Fig. 3A) mouse G4 mESC [28], mouse fibroblast-derived iPSCs [mFiPSC] [29], and hGriPSC (S2 Fig.) colonies on MEFs using the following protocol. Cultures were rinsed using Ca^2+^- and mG^2+^-free PBS and then treated with pre-warmed collagenase IV and Accutase as described above. To initiate differentiation of EBs, the mES media was replaced with EB media, consisting of DMEM-F12, 15% KOSR, 15% HI FBS, 1 mM L-glutamine, 0.1 mM 2-mercaptoethanol, and 1% Non-essential amino acids (NEAA, Invitrogen), 1% antibiotic-antimycotic solution (Invitrogen). EB media was used to resuspend the dissociated mGriPSC into a single-cell suspension. To minimize the presence of MEFs during EB formation, the cell suspension was placed to a gelatin-coated 100 mm culture plate in a 37°C, 5% CO2 incubator for 15–20 minutes. MEF cells typically adhere to gelatin faster than ESCs and iPSCs, thus the supernatant that is collected after 20 min contains fewer MEFs. This process was performed 2 times. Cell concentration was adjusted to approximately 6–10x10^5^ cells/ml using EB media. The suspension was then plated into ultra low-attachment 6-well plates (Corning, Tewksbury, MA) or 100mm plates coated with 2% poly-HEMA in ethanol which also achieves low-attachment. EBs were grown in suspension for 14 days, and subsequently attached to gelatin-coated plates for up to 30 days of additional culture. Half of the media in each well was collected and replaced daily with fresh EB media. This experiment was performed three times. Collected media was assayed for estradiol and progesterone by radioimmunoassay (RIA) at the Wisconsin National Primate Research Center (WNPRC) at the University of Wisconsin-Madison.

### Ovarian tissue markers

Markers of ovarian tissue were employed to characterize differentiation of mGriPSC. EBs from all three cell lines were also dissociated using pre-warmed 0.05% Trypsin-EDTA (GIBCO) and reattached to gelatin-coated plates as a cell monolayer for improved visualization of individual cells. After fixation, ICC for antigens characteristic of ovarian cell types, including anti-Müllerian hormone and receptor (AMH/R), follicle-stimulating hormone receptor (FSHR), aromatase (Cyp19a1), inhibin β-A (Inha), and estrogen receptor (ER), was performed both *in situ* and *in vitro* for ovarian granulosa cells and differentiated stem cells. In the adult female reproductive system, AMH, a member of the TGF-beta superfamily, localizes to the granulosa cells of the ovary and is a marker of ovarian follicle reserve. Furthermore, AMH is also believed to play a role in antral follicle recruitment. In this study we use this as a marker for ovarian tissue specificity. FSHR and ER are expressed by postnatal ovarian granulosa cells and are also involved in follicle recruitment and growth. Similarly, Inha is expressed by ovarian granulosa cells and is associated with secondary follicles. One of the primary ovarian functions is steroidogenesis regulated by aromatases. Cyp19a1 is a primary aromatase that converts 19-carbon androgens into 18-carbon estrogens. Additional ovarian markers used in this study include Foxl2, an ovarian tissue commitment marker, and Figla, a marker of germ cell lineage.

Differentiated stem cels that were positively-immunolabeled for these ovarian markers were counted in 4 fields of view per antibody per cell line, and the percentage of cells expressing each of the ovarian antigens was calculated for dissociated mGriPSC-EBs, G4 mESC-EBs, and mFiPSC-EBs. Undifferentiated cell colonies from all three cell lines were also stained for these ovarian antigens as a negative control (S4 Fig.).

### Statistical analyses

All endocirne data was analysed using a two-tailed T-test p<0.05 for significance. The microarray data were processed using the *lumi* package [34] for background subtraction, log2 transformation and quantile normalization. The R statistical environment was used to perform data analysis [http://www.r-project.org]. To filter out unresponsive probes, we removed probes that had detection *P*-values <0.01 (as determined by Illumina’s BeadStudio software) in less than or equal to one sample across all samples.

### Mouse primordial oocyte differentiation analysis

mGriPSCs and G4 mESCs were used to generate EBs, as described above, in defined media formulated by our lab to enrich ovarian cell types during differentiation (oocyte derivation media: DMEM, 15% KOSR, 15% HI ES-grade FBS, 2mM L-Glutamine, 1% NNEA, 0.1 nM 2-Mercaptoethanol, 100 U/ml ESGRO LIF, 5 ng/ml insulin, 0.01 U/ml PMSG, 50 μg/ml sodium pyruvate, 5 μg/ml Transferrin, 10 μg/ml Selenium, 50 ng/ml BMP-4, 5 μM Retinoic Acid, 5 μM Forskolin). After 2 weeks in suspension, EBs were dissociated and reattached to gelatin-coated plates, as described above, for an additional 10 days of culture as a monolayer of cells in oocyte derivation media. After fixation, ICC for mouse oocyte markers was performed with antibodies to Gdf9, Dazl, Boule, Mvh, and Zp1 (Santa Cruz; S1 Table). Positively-immunolabeled, spherical-shaped cells were counted in 7 fields of view and the percentage of cells expressing each of the oocyte antigens in both the dissociated mGriPSC-EBs and G4-EBs was calculated.

### Microarray mRNA expression analysis

Microarrays were run using the Illumina WG-6 v2.0 kit. cDNA was amplified from RNA extracted from cell lines or tissues as described above and run in triplicate. Our analyses compared suspended and attached mGriPSC- and mESC-EBs with mESC colonies, postnatal day 2 (P2) mouse ovaries, and isolated adult mouse GCs. The R statistical environment was used to perform data analysis (http://www.r-project.org). The microarray data were processed using the *lumi* package [34] for background subtraction, log2 transformation and quantile normalization. To filter out unresponsive probes, we removed probes that had detection *P*-values < 0.01 (as determined by Illumina’s BeadStudio software) in less than or equal to one sample across all samples (18 samples in total). The remaining 25,294 probes were used for analysis. If multiple probes represent a single gene, the probe with the highest median expression across all samples was chosen to represent the expression of that gene. The final dataset contains 18,029 genes. Heat maps were generated to illustrate hierarchal clustering of genome-wide expression as well as genes in the steroidogenic biosynthesis pathways. The set of core steroid biosynthesis enzymes was taken from a review paper [35]. Principal component analysis (PCA) was performed on the gene expression dataset using R’s *prcomp* function (S1 Materials). Differential expression analysis was performed using limma [36]. These were then evaluated to explore common differentially regulated genes (DRGs) and relevant gene regulatory networks (GRNs). Differential gene expression relevant to gonadogenesis and steroidogenesis was further analyzed using Ingenuity Pathways Analysis (IPA) software (Ingenuity Systems, Redwood City, CA).

### Genome-wide profiling of microRNA using next generation sequencing

Bar-coded multiplexed cDNA library preparation was performed as previously described [37]. For each sample, barcoded 3’ adapters were ligated to 2 μg of total RNA. Size fractionation during adapter ligations using radiolabeled 19- and 35-nucleotide markers were used to gel purify the miRNA fraction. Sequencing of amplified cDNA libraries was performed using 50 cycles of single-end sequencing by synthesis (Illumina HiSeq 2000). Barcodes were extracted and reads were aligned to the genome (hg19) and annotated using an in-house pipeline [37, 38]. The sum of all reads with fewer than 2 annotation mismatches was used to determine miRNA abundance, as previously described [39].

## Results

### Mouse ovarian GCs are readily reprogrammed into ovarian tissue-derived iPSCs

G4 mESCs were used as a standard for characteristic alkaline phosphatase reactivity and stem cell antibody ICC (Fig. 1A-D). The stem cell identity of the mGriPSC line as was verified by confirming positive alkaline phosphatase reactivity (Alk Phos, Fig. 1E) and expression of stem cell markers Oct4, SSEA-1, and Nanog (Fig. 1F-H). Colony morphology was comparable to mESCs. RT-PCR of ICC-confirmed Oct4, Nanog, and SSEA-1, as well as additional stem cell markers Gdf3 and Dnmt3b, was demonstrated in G4 mESCs and further verified pluripotency of the mGriPSCs (Fig. 1I). We confirmed that the mGriPSC line was consistently karyotypically normal by chromosome analysis, with 10 metaphase cells possessing 40 normal chromosomes (Fig. 1J). Additional culture of mGriPSC-EBs (Fig. 1L) in differentiation conditions resulted in generation of cell types from all three developmental germ layers as demonstrated by neurofilament (NF, ectoderm, Fig. 1M), smooth muscle actin (SMA, mesoderm, Fig. 1N) and alpha fetoprotein (αFP, endoderm, Fig. 1O) immunoreactivity. 100% of the six injected SCID mice formed a teratoma (Fig. 1P), which displayed differentiation into cells types of all germ layers [Fig. 1Q-S]. Collectively, these cytological characterization and functional differentiation studies confirm the successful generation of a novel ovarian tissue iPSC line.

### Characterization of endogenous stem cell gene expression in mGriPSCs

We interrogated activation of stem cell loci for Oct4, Sox2, cMyc and Klf4 in retrovirally reprogrammed mGriPSC (Fig. 1K). Our results demonstrate endogenous activation of all four genes reflecting successful reprogramming of our derived iPSC cells. PCR analysis with primers for the total transcripts [endogenous and transgene] confirmed presence of these genes in our iPS line, while transgene expression demonstrated various levels of silencing.

### Human GCs are also reprogrammable to generate hGriPSCs

Employing the same retroviral infection technique, we derived an analogous human granulosa cell-derived iPSC line and generated hGriPSC-EBs (S2A-D Fig.). The hGriPSC cultures morphologically resemble hESC colonies in that colonies are larger in diameter and flatter than mGriPSC colonies. Furthermore, they demonstrate expression of known human stem cell antigens OCT4, SSEA4, TRA-1–81, and NANOG by ICC analysis (S2E-H Fig.) and alkaline phosphatase reactivity (S2I Fig.). RT-PCR re-confirmed the expression of OCT4 and NANOG, as well as other stem cell markers DNMT3B and GDF3 (S2J Fig.). These results reliably verify the stem cell status of our human GC-derived iPSC line.

### Methylation analysis supports reprogramming and retention of GC signature

The methylation status of CpG islands #25–29 in the Oct4 gene was analyzed in mGriPSCs, G4 mESCs, and GCs (Fig. 2A). In mGriPSC, methylation of the CpG islands ranged from 32.7–48.8%, in mESCS 19.4–31.5%, and in GCs 61.4–85.7%. Average methylation was different in all three cells lines in the Oct4 gene (P≤0.001).

Methylation of the Foxl2 ovarian gene and Figla germ cell gene were not different in the pluripotent mGriPSCs and GC starting tissue (Fig. 2B-C; Foxl2 P = 0.84; Figla P = 0.606). Methylation of CpG islands #49–55 ranged from 0–5.2% in mGriPSC and 0–4.0% in GCs. Methylation of CpG islands #2–12 and #20–21 in the Figla gene ranged from 14.3–91.9% in mGripSC and 14.0–93.5% in GCs.

### mGriPSC EBs demonstrate preferential differentiation into estrogenic cell types

We compared steroid hormone production by EBs formed from mGriPSCs, mouse fibroblast-derived iPSCs (mFiPSCs), and G4 mESCs under identical culture conditions (mGriPSC: Fig. 3A). After 11 days, we detected estradiol (E2) at 344.1 pg/ml in the mGriPSC-EB conditioned media compared to 19.9 pg/ml and 28.1 pg/ml in the media of G4-EBs and mFiPSC-EBs, respectively. At the three assayed time points during the experiments, differentiating mGriPSC-EBs synthesized E2 at concentrations ranging from 5.3 to 17.3 times higher than those of the G4- or FiPSC-EBs (Fig. 3B). By contrast, G4-EBs produced primarily progesterone (P4), at a concentration of 1.95 ng/ml by day 11 in culture. mFiPSC-EBs made negligible E2 and P4 (Fig. 3C). Baseline RIA assay detected that “control,” unconditioned media contained 20.1 pg/ml of E2 and 0.04 ng/ml of P4. Our results not only demonstrate a significant disparity in the concentrations of the hormones produced, but also in the type of steroidogenic cells present in these cultures (estrogenic versus progestogenic).

### Differentiated mGriPSCS express ovarian and primitive germ cell antigens

**Fig 4:**
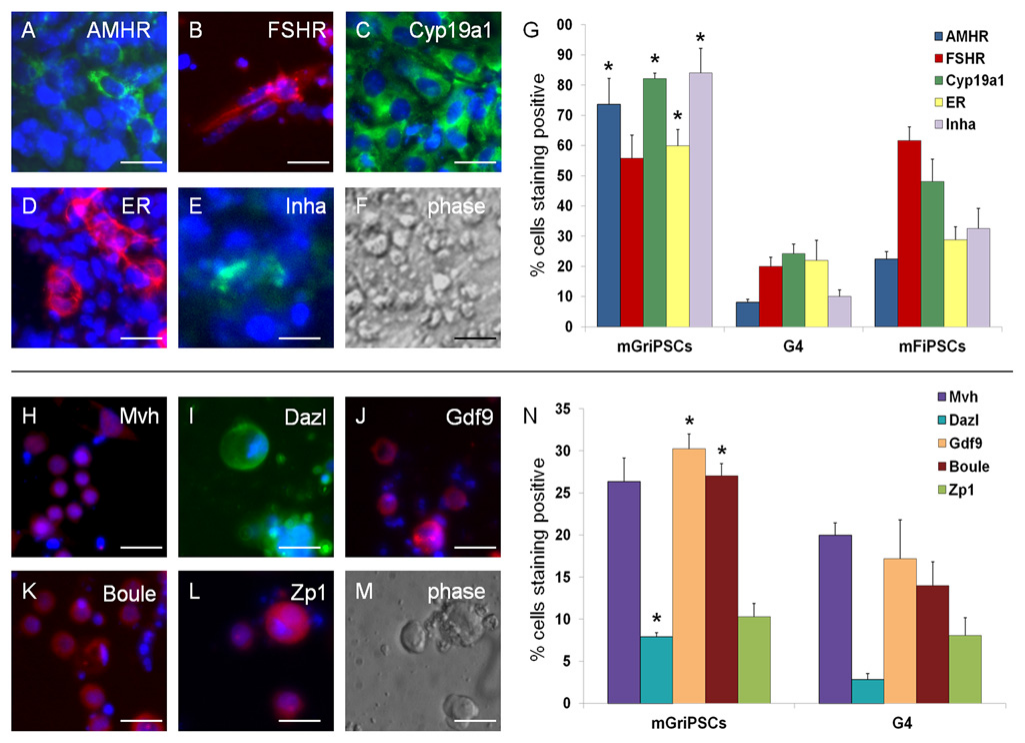
mGriPSC-EBs differentiate to express ovarian and primordial oocyte markers. Differentiating mGriPSC-EBs express ovarian markers AMHR **(A)**, FSHR **(B)**, Cyp19a **(C)**, Inha **(D)**, and ER **(E)** (phase contrast; **F)** at higher frequencies than G4- and mFiPSC-EB **(G)**. Expression of germ cell markers Mvh **(H)**, Dazl **(I)**, Gdf9 **(J)**, Boule **(K)**, and Zp1 **(L)** (phase contrast; **M**) are also increased in mGriPSC-EBs **(N).** Two-tailed t-test, *P < 0.05. Scale bars: **A-F** 10 μm; **H-M** 20 μm.

Distinct subpopulations of differentiated cells within the mGriPSC-EBs cultures expressed the ovarian markers AMHR, FSHR, Cyp19a1, ER, and Inhibin β-A (Inha; Fig. 4A-E). The average percent of positively-immunolabeled cells was calculated for EBs generated from the same three cell lines (mGriPSCs, FiPSCs, and G4 mESCs) cultured under identical conditions. AMHR, Cyp19a1, ER, and Inha were expressed in a larger proportion of cells in the mGriPSC-EB monolayered cultures compared to the other cell lines (Fig. 4G).

An increased cell population of cells within the differentiating mGriPSC-EBs also differentiated into cell types that expressed the primordial oocyte and germ cell antigens Mvh, Dazl, Gdf9, Boule, and Zp1 (Fig. 4H-L) compared to G4 mESC-EBs when cultured in identical, oocyte-enriching conditions. Compared to G4 mESC-EBs, the efficiency of primitive germ cell generation from mGriPSC was markedly improved, as evidenced by the 2.8-, 1.8-, and 1.9-fold fold expression of Dazl, Gdf9, and Boule, respectively (Fig. 4N). These results, along with the steroidogenic functionality of cultured mGriPSC-EBs, provide further support for the preferential homotypic differentiation of mGriPSCs into ovarian tissue.

### Genome-wide profiling of mRNA and microRNA supports the stem cell status of the mGriPSCs and hGriPSCs

#### Microarray analysis for stem cell markers

The stem cell genes Oct4, Nanog, SSEA-1, Gdf3 and Dnmt3b are found to be expressed in differentiating iPSCs comparable to corresponding G4 ESCs (S5A-E Fig.). This analysis further substantiates reprogramming of the GCs into pluripotent stem cells.

#### Microarray analysis of markers of ovarian tissue and gametogenesis

**Fig 5:**
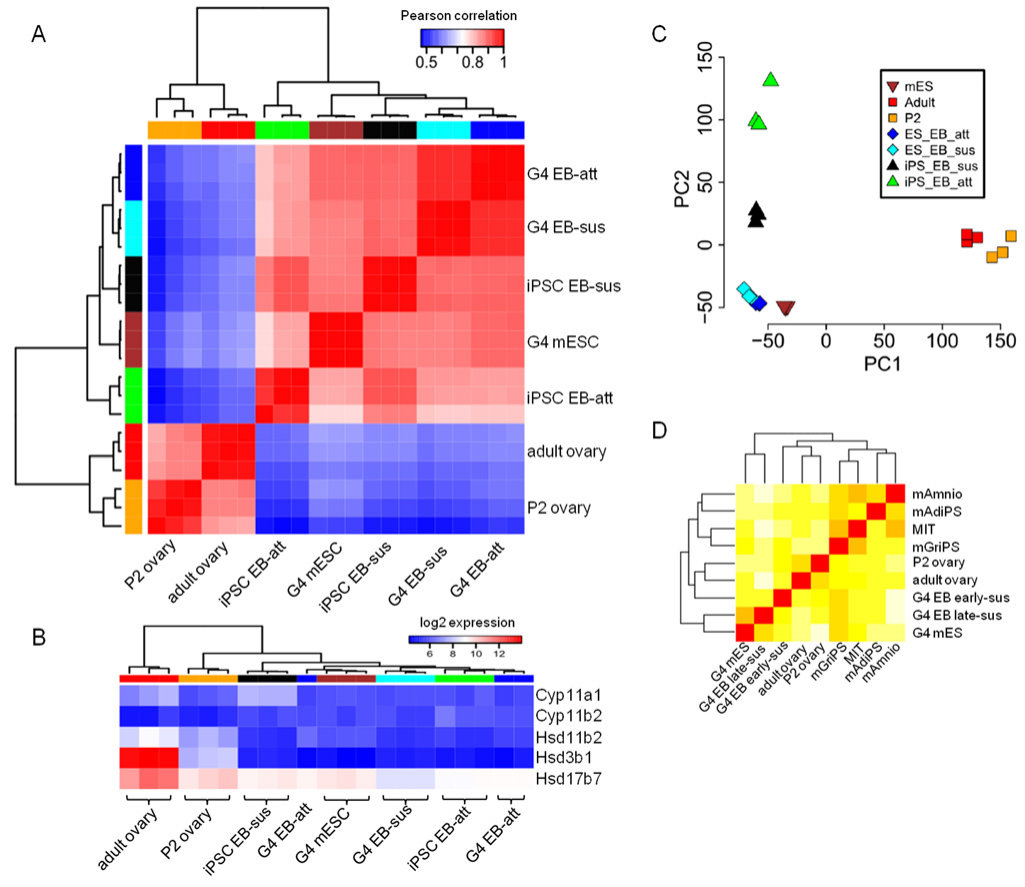
Genome-wide analysis of mRNA and microRNA confirms efficient pluripotent reprogramming of mGriPSCs and hGriPSCs. mGriPSC-EBs and mESC-EBs have comparable microarray expression profiles **(A)**. All ESC or EB samples show minimal steroidogenic mRNA compared to adult ovary samples **(B).** Principal component analysis (PCA) indicates attached and unattached mGriPSC-EBs are more disparate relative to the analogous mESC-EBs **(C)**. microRNA heatmaps support efficient stem cell reprogramming of mGriPSCs **(D)**.

We investigated the gene expression patterns that coincide with the initiation of steroidogenesis, a primary function of the mammalian ovary, in our estrogen-producing mGriPSC-EBs and in the adult mouse ovary compared to suspended mGriPSC-EBs, suspended mESC-EBs, and the immature, postnatal day 2 (P2) mouse ovary (Fig. 5A-C, S5F-K Fig.). We computed genome-wide Pearson correlation coefficient of every pair of samples in the microarray dataset (Fig. 5A). We found two main clusters: [i] P2 and Adult ovary, and [ii] mES and EBs. This indicates that regardless of the source of EB (mESC or from iPSC); suspended or attached, their genome-wide expression profiles more closely resemble that of mESC colonies than that of postnatal primary tissue. We further analyzed the expression patterns of key genes that encode enzymes in the steroid hormone biosynthesis pathways. There is a clear increase in expression of these genes in adult ovary compared to P2, but the expression in other EB and ES cells are generally lower (Fig. 5B-C, S5H Fig.).

Principal Component Analysis (PCA) was performed to identify important axis of variability among the samples. PC1 and PC2 collectively account for 61% of the variability among the samples. PC1 separates primary tissues (including P2 and Adult ovary) from EB and mES cells; PC2 separates iPSC-derived EBs from mESC-derived EBs (Fig. 5C). The difference between suspended and attached iPSC-derived EBs is much larger than the analogous difference between suspended and attached ESC-derived EBs.

Additionally, microarray analysis confirms expression of ovarian genes as well as markers of gametogenesis (S5L-P Fig.) supporting our ICC results.

#### MicroRNA analysis

Parallel analyses of the mGriPSCs were performed utilizing microRNA sequencing and expression analysis. In the initial analysis, a comparison of mGriPSCs microRNA to that of mESCs and the originating somatic GC cells was performed. Our results revealed a closer relationship between mGriPSCs and mESCs in terms of genome-wide analysis of stem cell profiles than with other tissue types such as GCs (Fig. 5D). We performed a similar microRNA profiling analysis for hGriPSCs, and also showed that hGriPSCs are much more similar to hESCs than ovarian cells (S2K Fig.), further supporting the stem cell status of our hGriPSCs.

## Discussion

Since the initial discovery and isolation of mESCs in 1981 [40] and the subsequent isolation of hESCs in 1998 [41], we have learned much about stem cell development and their differentiation towards various cell fates. These breakthroughs have led to the recent development of methods to produce somatic cell-derived, induced pluripotent stem cells (iPSCs) [2, 42]. Similar to hESCs, iPSCs hold therapeutic potential in regenerative medicine for the treatment of cell-based diseases such as Parkinson’s, diabetes, and premature ovarian failure. A distinct advantage of iPSCs is that these are patient-specific and therefore antigenically match the donor somatic cell type and provide a platform for developing autologus therapies. In this study, we examine a novel iPSC population that we have generated using discarded ovarian tissue, specifically the granulosa cells of the ovarian follicle. These cells are an available by-product of oocyte harvesting from patients undergoing IVF and embryo transfer. We propose an original and intriguing approach to the future treatment of cell-based diseases using homotypically-derived iPSCs from the desired tissue type.

**Fig 6:**
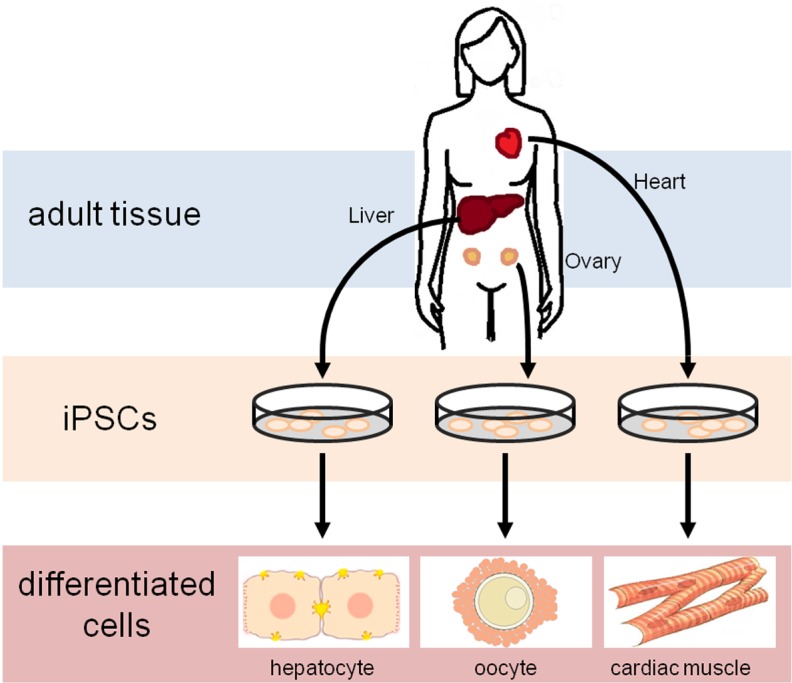
An autologous iPSC model in regenerative medicine. Terminally-differentiated cells from various adult organs may generate iPSC lines for homotypic differentiation into desired tissues.

The power of iPSC generation lies in the ability to make these cells from a variety of different tissues. This is especially advantageous because cells or tissues that are routinely discarded during normal surgical treatment or patient care can be used for iPSC generation. For example, adipocytes might be obtained during liposuction, amniocytes from amniocentesis, and, in this study, GCs from infertility treatment. There are over 140,000 IVF cycles annually in the U.S. [25] which provide an abundant source of terminally-differentiated, adult ovarian cells for derivation of iPSCs. Thus the need for embryonic tissue is minimized. Moreover, these cells are donor-specific and thereby transcend a significant hurdle to future applications in transplant medicine and cell-based therapies (Fig. 6).

While it seems possible to produce iPSCs by reprogramming nearly any somatic cell, we increasingly understand subtle but important differences between each iPSC line. Some of these variations are largely related to the originating type of tissue and inherent plasticity of the somatic cells. For example, iPSCs are readily produced from adult neural stem cells with as few as 2 reprogramming factors, while amniocytes require all 4 factors; yet amniocytes generate iPSCs with an efficiency that is orders of magnitude higher than other cell types. Similarly, human foreskin cells, which are developmentally “young,” are an efficacious source of somatic cells for iPSC derivation [31, 42, 43]. The difference in derivation efficiency and number of reprogramming genetic vectors required to completely reprogram these somatic cells demonstrates intrinsic disparities in the derived iPSCs. The exact differences are beginning to emerge upon closer study of their differentiation properties. Mounting evidence indicates that iPSC differentiation capacity is largely affected by cell line epigenetic signature [5, 9–11, 13, 14].

We hypothesized that the epigenetic memory of mGriPSCs would influence spontaneous differentiation such that they would be more likely to revert to homotypic, differentiated cell types. This assumption was based on the findings of several reports that observed biased differentiation of other iPSCs towards their originating tissue types [6, 10, 11, 14, 15]. It appears that the epigenetic memory of iPSCs alters the likelihood of differentiation into different cells types. While the iPSCs are indeed pluripotent, the epigenetic variations may promote biased differentiation toward the originating tissue type. We proposed that this characteristic could be beneficial if the iPSCs are derived from the desired tissue type. Thus we investigated the effects of epigenetic memory by comparing differentiation competency of the ovarian-iPSCs with that of mESCs or other unrelated mouse iPSC (mFiPSCs).

In support of our hypothesis, we demonstrate that mGriPSCs readily differentiate into homotypic ovarian cells types, such as the steroidogenic cells, under specific conditions. Estradiol, which is produced by the ovary *in vivo*, was detected in mGriPSC-conditioned media at physiologic, ovulatory concentrations and was synthesized at levels much higher than those of the mESC and mFiPSC lines. Additionally, the increased expression of Cyp19a1 (aromatase), which converts testosterone to estradiol, in the mGriPSCs culture is consistent with the emergence estrogenic cells. Significantly lower frequency of Cyp19a1-positive cells in the other cell types may relate to the decreased estradiol synthesis detected in those cultures. Preliminary IPA pathway analysis using previously-described tissue-specific networks (see S6 and S7 Figs.) reflects these steroidogenesis- and gonadogenesis-centric, genetic relationships in our cultured cell population, which likely contributes to this predisposition. In the normal menstrual cycle, follicle growth and oocyte maturation occurs during the follicular phase. During this period of development, the granulosa cells in the follicle synthesize estradiol [24, 44]. It is only after ovulation of a mature oocyte and loss of the close physical association between the granulosa cells and the oocyte that granulosa cells are luteinized and begin to produce progesterone in higher amounts. Therefore, it is interesting that mGriPSC-EBs showed a bias towards estradiol synthesis. This level and specificity of steroidogenesis is not reflected by the other stem cell populations used in these comparative analyses. Thus the mGriPSCs hold particular potential for therapeutic applications in restoration of ovarian tissue.

Furthermore, the subpopulations of the mGriPSC culture shifting towards the gamete cell fate is increased in mGriPSC differentiation, as evidenced by the higher incidence of oocyte and germ cell antigen expression. IPA pathway analysis again identified known gene networks present in our mGriPSC cultures that related to gametogenesis regulation (S8 Fig.). These findings are consistent with our hypothesis, considering that the two primary functions of the ovary, steroidogenesis and gametogenesis, are realized in the differentiation behavior of the mGriPSC line.

A close evaluation of mGriPSCs and hGriPSCs using microarray and microRNA analyses further confirms that reprogramming of GCs into iPSCs was successful. While there is microarray evidence of slight increased steroidogenic activity compared to mESC colonies, the relatively low proportion of steroidogenic and ovarian genes measured in our microarrays, compared to other somatic tissue and stem cells genes, likely indicate that the population of steroid-expressing cells is relatively small compared to all the EB-derived cell types. A limitation of our study is that heterogeneity may be obscuring unique gene profiles relevant to our desired, differentiated tissue type. Regardless, the microarray analyses support differentiation of iPSCs along ovarian commitment pathways and demonstrates differentiation of oocyte-specific genes such as Zp1. Ongoing studies in our laboratory are addressing the issue of heterogeneity by attempting to isolate pure populations of differentiated cells for further analysis.

These provocative results are exciting and provide a platform for tissue-specific differentiation in regenerative medicine by taking advantage of the preferential differentiation of homotypic tissue. Current studies demonstrate that not only is it difficult to direct stem cells’ differentiation, but also often incomplete, precluding transplantation of mixed, differentiated stem cell cultures for fear of generating tumors from undifferentiated cells. Additionally, low differentiation efficiency makes it difficult to obtain adequate number of ovarian-lineage cells [45]. Other studies attempting to generate granulosa-like cells for ESCs or iPSCs add growth factors [46] or employ a co-culture system, differentiating stem cells alongside functional GCs [47]. This would not be a viable option for future applications to POF patients. Instead, we chose to exploit the iPSCs alleged inherent bias during differentiation. While preferential differentiation might not completely obviate the concerns of heterogeneity and low differentiation efficiency, the percentage of desired cells could be enriched and hopefully purified prior to use.

While the use of retroviral vectors provides an efficient vehicle for the transduction of targeted cells, retroviruses do randomly integrate into DNA and could induce some genomic instability. Although it appears that deleterious mutations may be selected out during sequential subcultures of iPSCs, any clinical application of these results would require stable reprogramming of cells with non-integrating or site-specific transformation vectors [48–50]. Furthermore, here we demonstrate activation of endogenous stem cell genes with varying degrees of retroviral transgene silencing. Additionally, we have passaged these cells for the last two years with reliable maintenance of stem cell properties. It has been previously reported and well-documented that retroviral expression is rapidly silenced in mammalian cells [51]. We are currently exploring such approaches as it pertains to the reprogramming of GC cells.

It has become evident that using completely undifferentiated cells to form desired cell types is a daunting task. Thus, current research is also looking to better understand the mechanisms of successful transdifferentiation [52–54], which involves conversion of one terminally-differentiated cell type into another without a pluripotent intermediate. It has been shown that a mutation in the sex-determining *Wnt* pathway during development can lead to pre-granulosa cells transdifferentiating from female to male gonad cell types [55]. Other studies reinforce the notion that GCs may in fact have considerable plasticity [56, 57]. In our study, perhaps some of the improved efficiency of homotypic differentiation is a reflection of epigenetic memory and histone methylation mechanisms, which are currently being investigated in the context of transdifferentiation for many cell types [58–60]. It is possible that tissue-specific epigenetic programming allows differentiated cells to more effectively pursue epigenetically-biased differentiation pathways. Further studies in our laboratory are investigating these areas as well as other approaches to enrich and isolate specific differentiated subpopulations of cells. Our goal is to facilitate preparation of these cells for *in vivo* transplant studies using a mouse model.

## Conclusion

Tissue-specific iPSCs, such as our ovarian GC-derived iPSCs, show biased functional differentiation into the originating tissue type, supporting the hypothesis for epigenetic-mediated mechanisms of homotypic differentiation of iPSCs. This approach may prove useful when applied to specific targeted tissue derivation for use in cell-based therapies.

## Supporting Information

S1 Fig Experimental plan for investigation of epigenetic memory in an iPSC line, comparing our novel mGriPSC line with G4 mESCs for epigenetic bias by assessing spontaneous differentiation, functionality, and gene expression profiles. (TIF)

S2 Fig Derivation of hGriPSCs from discarded human GCs. Anonymous, discarded human GCs **(A)** are infected with retroviral vectors and produce primitive hGriPSC colonies in approximately 10 days **(B)**. Purification allows generations of mature hGriPSC colonies **(C)** and hGriPSC-EBs **(D)**. Presumptive human GriPSCs colonies demonstrate hESC immunogenicity for stem cell markers OCT4 **(E)**, SSEA4 **(F)**, TRA-1–81 **(G)**, and NANOG **(H)**. Colonies are also alkaline phosphatase reactive **(I)**. Additionally, RT-PCR confirms hGriPSC expression of stem cell genes OCT4, NANOG, DNMT3B, and GDF3 **(J)**. MicroRNA analysis supports reprogramming of the human GC-derived iPSC line **(K)**. Scale bars: **A-B** 50 μm; **C-D** 250 μm; **E-I** 100 μm.
(TIF)

S3 Fig Absence of stem cell marker expression in primary granulosa cells. Harvested granulosa cells were cultured *in vitro* for 1 day and stained with stem cell antigens Oct4 **(A)**, Nanog **(B)** and SSEA-1 **(C)**. Sectioned mouse ovarian follicles demonstrated positive AMHR **(D)** and aromatase (Cyp19a1; **E)** expression. Scale bars: 50 μm.
(TIF)

S4 Fig Absence of pre-existing ovarian cell markers expression in mouse stem cell lines. After verification of pluripotency **(A,H,O)**, all mouse cell lines, including G4 mESCs, newly-derived mGriPSCs, and mFiPSCs, were immunostained for ovarian cell markers AMHR **(B,I,P)**, Cyp19a1 **(C,J,Q)**, inhibin (inha; **D,K,R)** and germ cell markers Mvh **(E,L,S)**, Dazl **(F,M,T)**, and Zp1 **(G,N,U)**. Scale bars: 200 μm.
(TIF)

S5 Fig Microarray analysis of specific stem cell markers, ovarian markers, and gametogenesis markers. Stem cell gene expression is consistent with that of mESCs **(A-E)** and supports successful reprogramming. Expression of genes involved in ovarian development and function **(F-K)**, steroidogenesis **(H)** and gametogenesis **(L-P)** are expressed at lower levels in mGriPSC compared to adult ovarian tissue, but is again consistent with mESCs.
(TIF)

S6 Fig Estradiol-regulated IPA pathway. Previously described regulatory networks involving estradiol synthesis were represented in the preliminary mRNA analysis of the mGriPSC-EB culture *in vitro*. *P* ≤ 0.05, false discovery rate (FDR) = 0.10, and fold change cutoff = 1.5.
(TIF)

S7 Fig Gonadogenesis pathway represented in mGriPSC culture. mRNA analyses of the mGriPSC-EB culture demonstrated the expression of known gonadogenesis gene networks. *P* ≤ 0.05, false discovery rate (FDR) = 0.10, and fold change cutoff = 1.5.
(TIF)

S8 Fig Gametogenesis pathways represented in mGriPSC culture. mRNA analyses of the mGriPSC-EB culture demonstrated expression of components **(A-C)** of previously-determined gametogenesis gene networks. *P* ≤ 0.05, false discovery rate (FDR) = 0.10, and fold change cutoff = 1.5.
(TIF)

S1 Materials (DOCX)

S1 Table Immunocytochemistry antibodies. (DOCX)

S2 Table PCR Primer Sequences. (DOCX)
