# Supplementary figures and images for: Efficient Differentiation of Steroidogenic and Germ-Like Cells from Epigenetically-Related iPSCs Derived from Ovarian Granulosa Cells
Source: PLoS One. 2015 Mar 9;10(3):e0119275. doi: 10.1371/journal.pone.0119275 (PMC4353623; doi:10.1371/journal.pone.0119275)

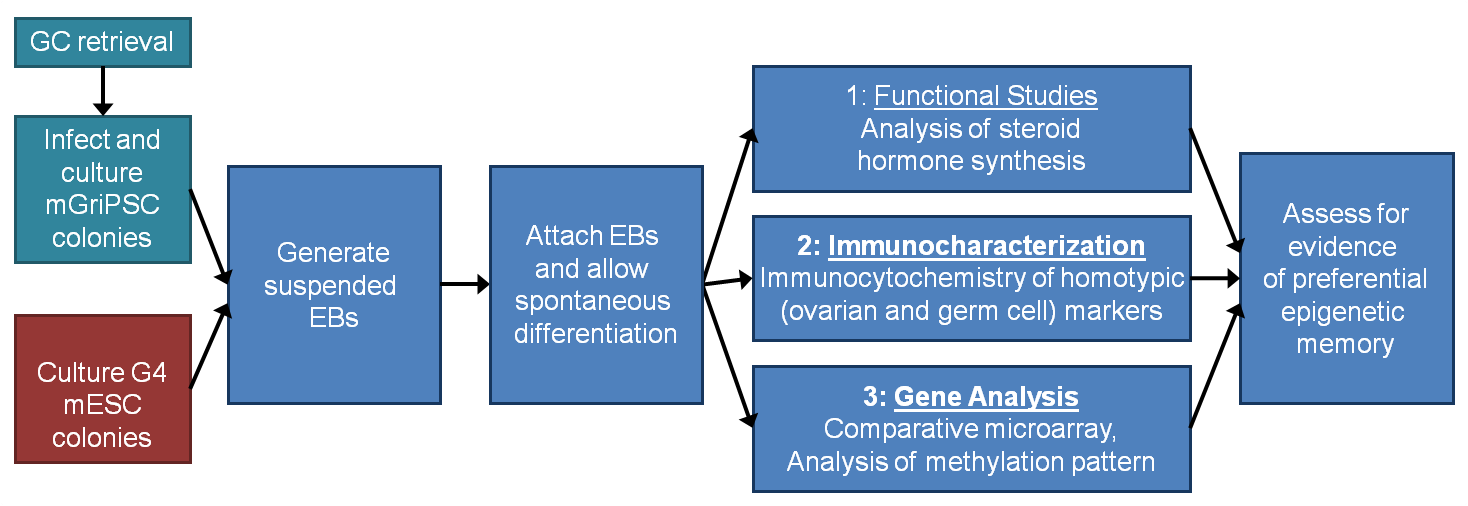

Supplement: S1 Fig — (TIF) [file pone.0119275.s001.tif]

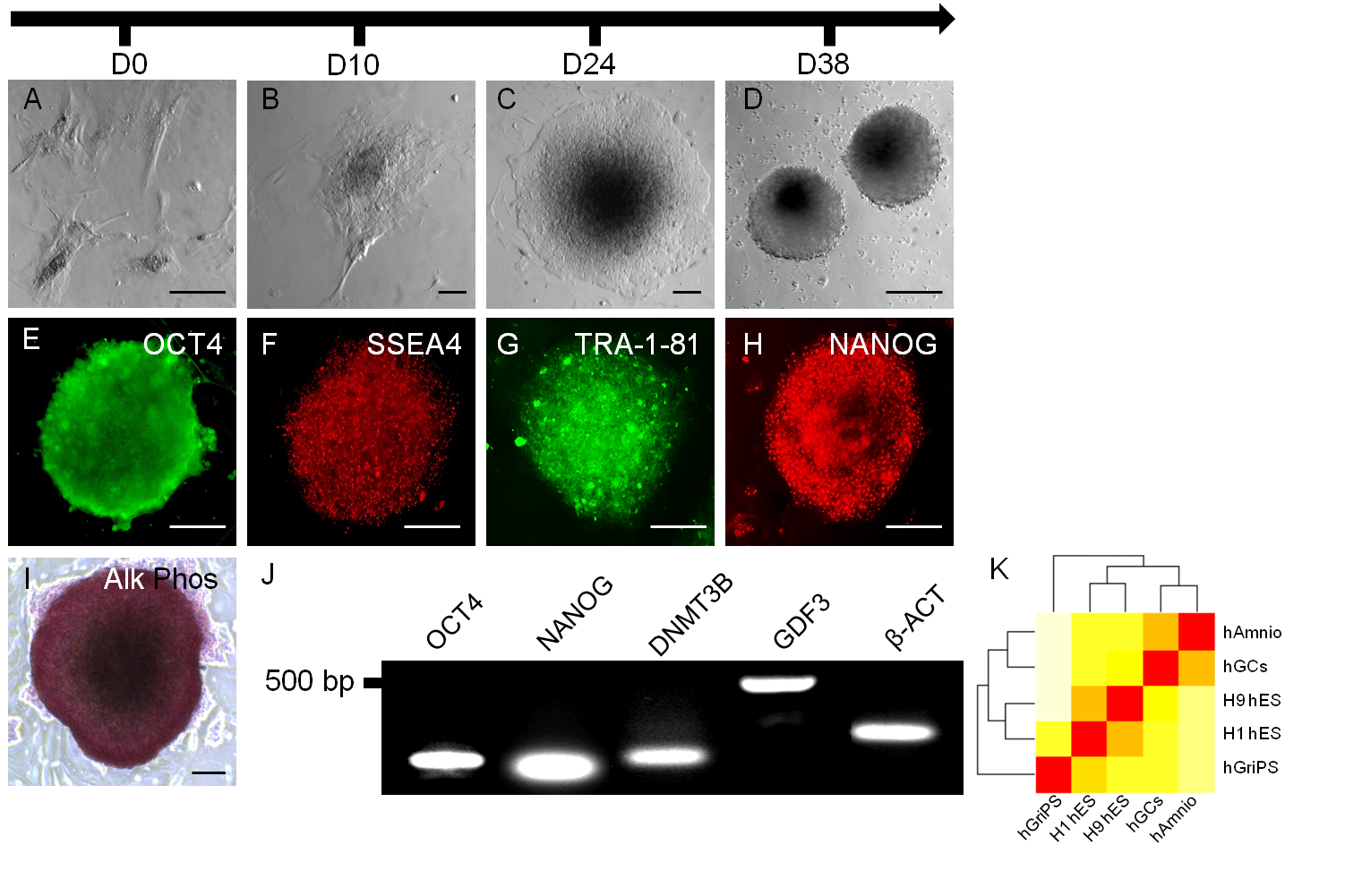

Supplement: S2 Fig — Anonymous, discarded human GCs (A) are infected with retroviral vectors and produce primitive hGriPSC colonies in approximately 10 days (B). Purification allows generations of mature hGriPSC colonies (C) and hGriPSC-EBs (D). Presumptive human GriPSCs colonies demonstrate hESC immunogenicity for stem cell markers OCT4 (E), SSEA4 (F), TRA-1–81 (G), and NANOG (H). Colonies are also alkaline phosphatase reactive (I). Additionally, RT-PCR confirms hGriPSC expression of stem cell genes OCT4, NANOG, DNMT3B, and GDF3 (J). MicroRNA analysis supports reprogramming of the human GC-derived iPSC line (K). Scale bars: A-B 50 μm; C-D 250 μm; E-I 100 μm. (TIF) [file pone.0119275.s002.tif]

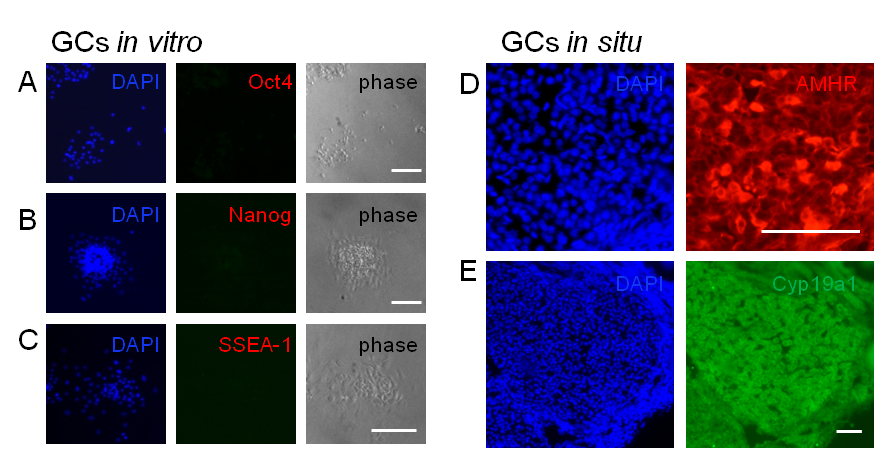

Supplement: S3 Fig — Harvested granulosa cells were cultured in vitro for 1 day and stained with stem cell antigens Oct4 (A), Nanog (B) and SSEA-1 (C). Sectioned mouse ovarian follicles demonstrated positive AMHR (D) and aromatase (Cyp19a1; E) expression. Scale bars: 50 μm. (TIF) [file pone.0119275.s003.tif]

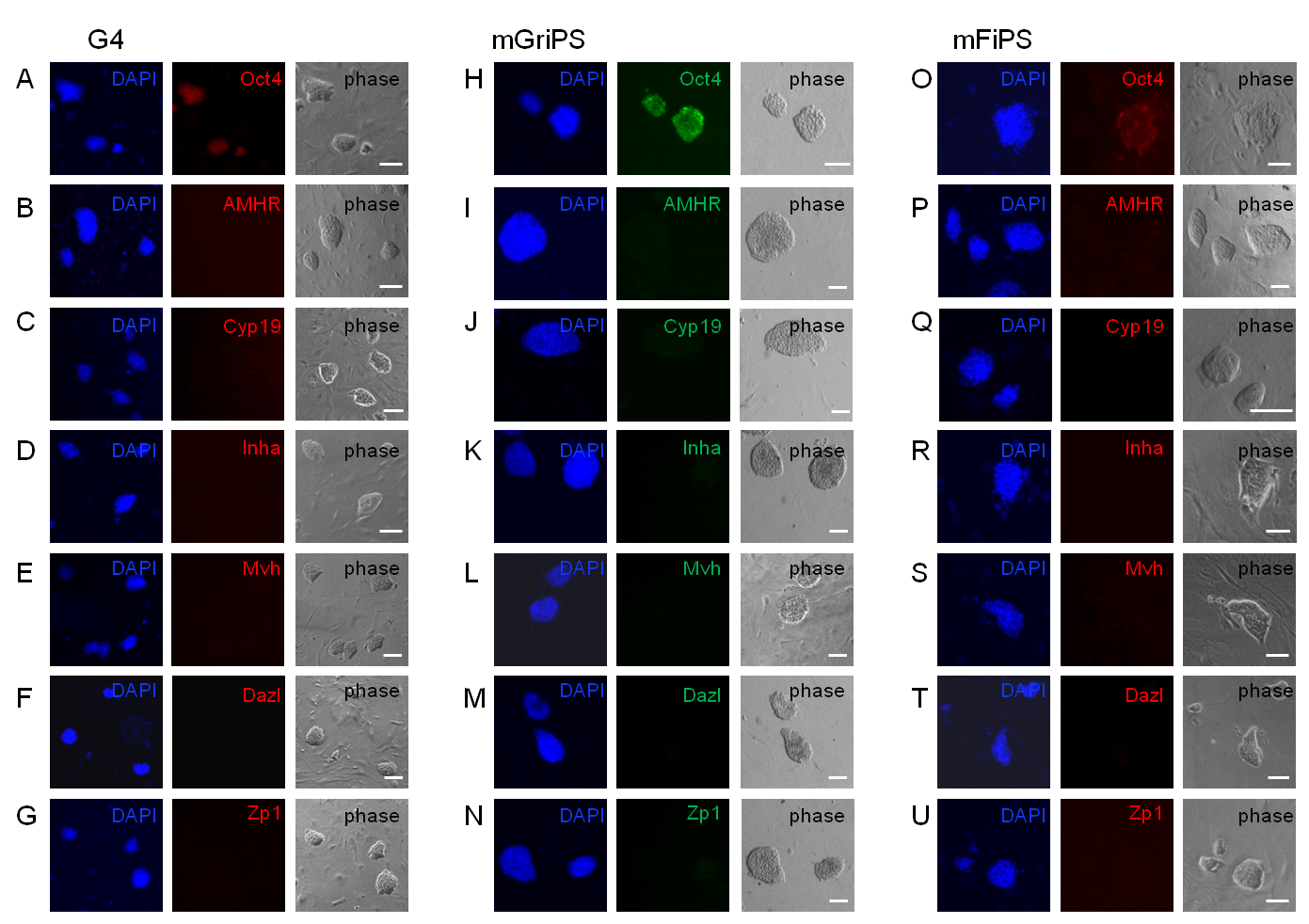

Supplement: S4 Fig — After verification of pluripotency (A,H,O), all mouse cell lines, including G4 mESCs, newly-derived mGriPSCs, and mFiPSCs, were immunostained for ovarian cell markers AMHR (B,I,P), Cyp19a1 (C,J,Q), inhibin (inha; D,K,R) and germ cell markers Mvh (E,L,S), Dazl (F,M,T), and Zp1 (G,N,U). Scale bars: 200 μm. (TIF) [file pone.0119275.s004.tif]

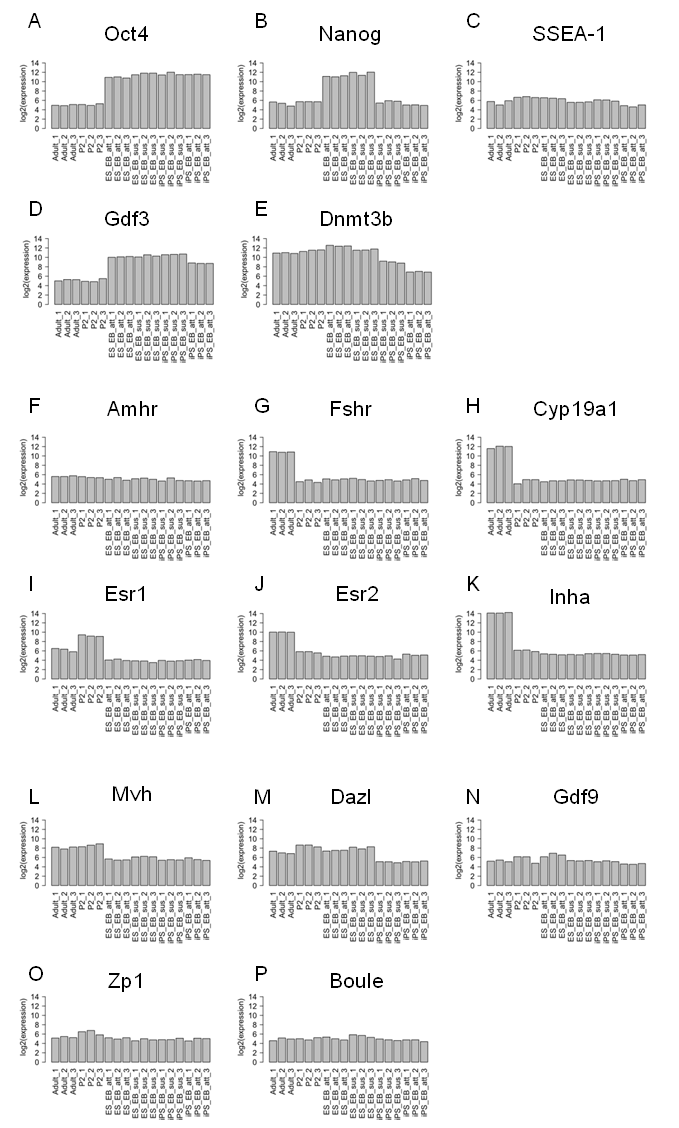

Supplement: S5 Fig — Stem cell gene expression is consistent with that of mESCs (A-E) and supports successful reprogramming. Expression of genes involved in ovarian development and function (F-K), steroidogenesis (H) and gametogenesis (L-P) are expressed at lower levels in mGriPSC compared to adult ovarian tissue, but is again consistent with mESCs. (TIF) [file pone.0119275.s005.tif]

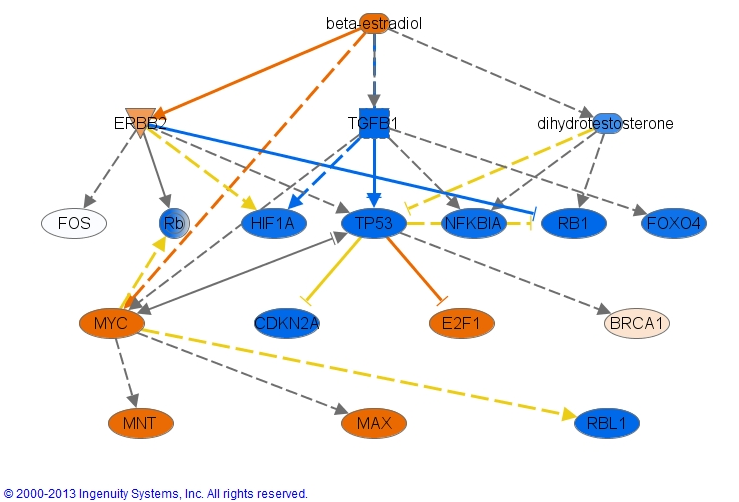

Supplement: S6 Fig — Previously described regulatory networks involving estradiol synthesis were represented in the preliminary mRNA analysis of the mGriPSC-EB culture in vitro. P ≤ 0.05, false discovery rate (FDR) = 0.10, and fold change cutoff = 1.5. (TIF) [file pone.0119275.s006.tif]

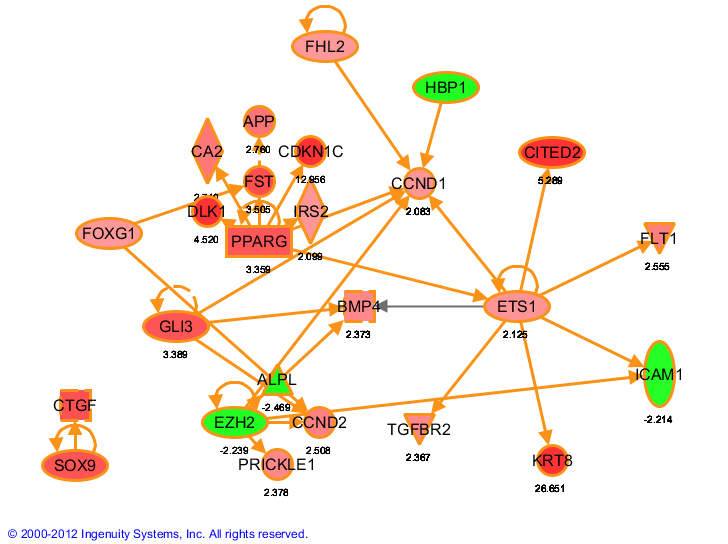

Supplement: S7 Fig — mRNA analyses of the mGriPSC-EB culture demonstrated the expression of known gonadogenesis gene networks. P ≤ 0.05, false discovery rate (FDR) = 0.10, and fold change cutoff = 1.5. (TIF) [file pone.0119275.s007.tif]

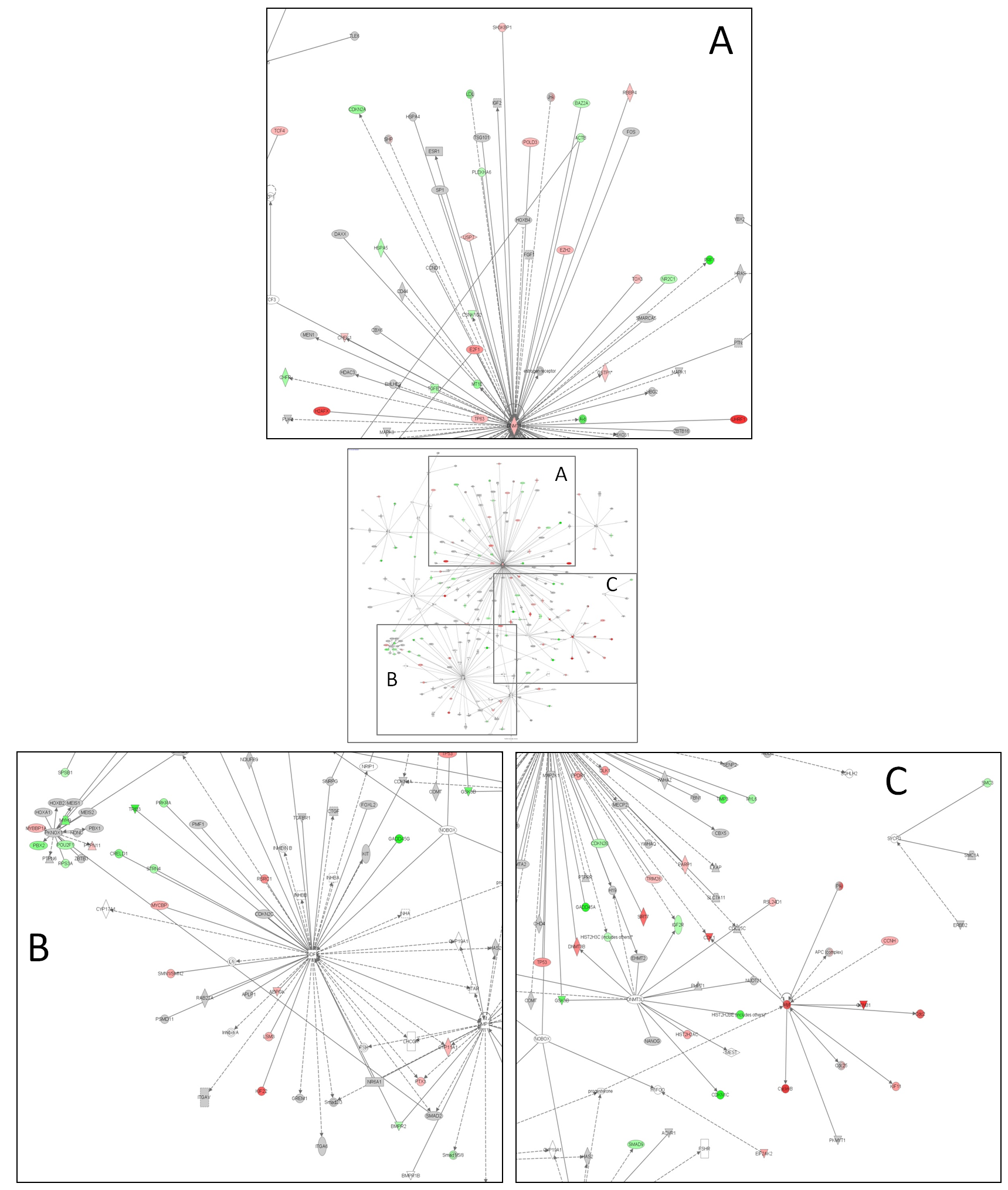

Supplement: S8 Fig — mRNA analyses of the mGriPSC-EB culture demonstrated expression of components (A-C) of previously-determined gametogenesis gene networks. P ≤ 0.05, false discovery rate (FDR) = 0.10, and fold change cutoff = 1.5. (TIF) [file pone.0119275.s008.tif]
