# Supplementary material for: Efficient Differentiation of Steroidogenic and Germ-Like Cells from Epigenetically-Related iPSCs Derived from Ovarian Granulosa Cells
Source: PLoS One. 2015 Mar 9;10(3):e0119275. doi: 10.1371/journal.pone.0119275 (PMC4353623; doi:10.1371/journal.pone.0119275)
Supplement: S1 Materials — (DOCX) [file pone.0119275.s009.docx]

**SUPPLEMENTAL MATERIALS AND METHODS**

**Microarray and Ingenuity Pathway Analysis (IPA).**

We performed a preliminary interrogation of previously-defined gene sets for evidence of gametogenesis (oocytes), steroidogenesis (estradiol), and gonadogenesis (ovary) pathways using Illumina platform microarray with Gene Set Enrichment Analysis (GSEA) and IPA. There were 9204 analysis-ready genes. After applying selective parameters (*P*-value ≤0.05, false discovery rate (FDR) = 0.10, and fold change cutoff = 1.5) there were a total of 3242 differentially regulated genes (DRGs).  1792 DRGs were upregulated and 1452 DRGs were down regulated in the attached mGriPSC-EBs. The analysis included observed genes with strongly- and moderately-predicted relationships in order to elucidate any possible relationships between genes, molecules, and networks in the abovementioned gene regulatory pathways. While several related genes are identified, due to heterogeneity of the differentiating stem cell population, only few were significantly noted to be differentially expressed. Of particular interest were DNMT1, H2AFX, and XIST, which are all related to gametogenesis.
