# Supplementary material for: Efficient Differentiation of Steroidogenic and Germ-Like Cells from Epigenetically-Related iPSCs Derived from Ovarian Granulosa Cells
Source: PLoS One. 2015 Mar 9;10(3):e0119275. doi: 10.1371/journal.pone.0119275 (PMC4353623; doi:10.1371/journal.pone.0119275)
Supplement: S1 Table — (DOCX) [file pone.0119275.s010.docx]

**SUPPLEMENTAL TABLES**

**Table S1. Immunocytochemistry antibodies.**

| **Antibody** | | **Company** | **Cat. #:** |
| --- | --- | --- | --- |
| SSEA-1 | Millipore | | MAB4301 |
| SSEA-4 | Millipore | | MAB430 |
| Oct4 | Abcam | | Ab18976 |
| Tra-1-60 | Millipore | | MAB4360 |
| Tra-1-81 | Millipore | | MAB4381 |
| Nanog | Abcam | | Ab106465 |
| AMHR | Abcam | | Ab64762 |
| NF | Abcam | | Ab24575 |
| SMA | Abcam | | Ab5694 |
| αFP | Santa Cruz | | Sc-8108 |
| FSHR | Santa Cruz | | Sc-7798 |
| Cyp19a1 | Abcam | | Ab35604 |
| INH β-A | Santa Cruz | | Sc-166503 |
| ER | Abcam | | Ab3577 |
| MVH | Abcam | | Ab13840 |
| DAZL | Abcam | | Ab34139 |
| BOULE | Abcam | | Ab104491 |
| GDF9 | Abcam | | Ab93892 |
| ZP1 | Santa Cruz | | Sc-23706 |
