# Supplementary material for: Efficient Differentiation of Steroidogenic and Germ-Like Cells from Epigenetically-Related iPSCs Derived from Ovarian Granulosa Cells
Source: PLoS One. 2015 Mar 9;10(3):e0119275. doi: 10.1371/journal.pone.0119275 (PMC4353623; doi:10.1371/journal.pone.0119275)
Supplement: S2 Table — (DOCX) [file pone.0119275.s011.docx]

| **Table S2. PCR Primer Sequences.** | | | | |
| --- | --- | --- | --- | --- |
| **Gene** | **Accession No.** | **Product Length** | **Forward Primer** | **Reverse Primer** |
| **mOct4** | NM_013633 | 612 bp | 5’-CACGAGTGGAAAGCAACTCA-3’ | 5’-CTGGGAAAGGTGTCCCTGTA-3’ |
| **mNanog** | NM_028016 | 223 bp | 5’-CAGGTGTTTGAGGGTAGCTC-3’ | 5’-CGGTTCATCATGGTACAGTC-3’ |
| **mGdf3** | NM_008108 | 164 bp | 5’-ACCTTTCCAAGATGGCTCCT-3’ | 5’-CCTGAACCACAGACAGAGCA-3’ |
| **mDnmt3b** | NM_001003961 | 165 bp | 5’-GCGCAGCGATCGGCGCCGGAGAT-‘3 | 5’-CATACCCGGTGGCACCCTGTTCTTC  AGTCA-3’ |
| **mOct4 endo** | NM_013633 | 221 bp | 5’-TCGGGGTGCCCACCTTC-3’ | 5’-CCCTCCGCAGAACTCGTATG-3’ |
| **mOct4 trans** | -- | 192 bp | 5’-CATCCTCTAGACTGCCGGA-3’ | 5’-TGGAAGCTTAGCCAGGTTCG-3’ |
| **mOct4 total** | -- | 140 bp | 5’-AGCGAACCAGTATCGAGAAC-3’ | 5’-TTACAGAACCACACTCGGAC-3’ |
| **mSox2 endo** | NM_011443 | 227 bp | 5’-CATCCCAATTGCACTTCGCC-3’ | 5’-gcttcagctccgtctccatc-3’ |
| **mSox2 trans** | -- | 233 bp | 5’-CATCCTCTAGACTGCCGGA-3’ | 5’-ccgggaccataccatgaagg-3’ |
| **mSox2 total** | -- | 126 bp | 5’-AGCTACAGCATGATGCAGGA-3’ | 5’-GGTCATGGAGTTGTACTGCA-3’ |
| **mcMyc endo** | [NM_001177352](http://www.ncbi.nlm.nih.gov/nuccore/NM_001177352.1) | 150 bp | 5’-CGCGATCAGCTCTCCTGAAA-3’ | 5’-GCTGTACGGAGTCGTAGTCG-3’ |
| **mcMyc trans** | -- | 163 bp | 5’-GGGGTGGACATCCTCTAGACT-3’ | 5’-ATAGGGCTGTACGGAGTCGT-3’ |
| **mcMyc total** | -- | 179 bp | 5’-ACTCTGAGGAGGAACAAGAA-3’ | 5’-TGGAGACGTGGCACCTCTT-3’ |
| **mKlf4 endo** | [NM_010637](http://www.ncbi.nlm.nih.gov/nuccore/NM_010637.3) | 220 bp | 5’-TTCGTTGACTTTGGGGCTCG-3’ | 5’-AGAGAGTTCCTCACGCCAAC-3’ |
| **mKlf4 trans** | -- | 138 bp | 5’-GCCGCCCCCTTCACC-3’ | 5’-GTGAGAGAGTTCCTCACGCC-3’ |
| **mKlf4 total** | -- | 105 bp | 5’-TCTCAAGGCACACCTGCGAA-3’ | 5’-TAGTGCCTGGTCAGTTCATC-3’ |
| **β-actin** | NM_007393 | 236 bp | 5’-TGTTACCAACTGGGACGA  CA-3’ | 5’-CCATCACAATGCCTGTGG  TA-3’ |
| **hOct4** | NM_001173531 | 144 bp | 5’-GACAGGGGGAGGGGAGGAG  CTAGG-3’ | 5’-CTTCCCTCCAACCAGTTGCC  CCAAAC-3’ |
| **hNanog** | NM_024865 | 213 bp | 5’-TTCCTTCCTCCATGGATCTG-3’ | 5’-TCTGCTGGAGGCTGAGGTAT-3’ |
| **hDnmt3b** | NM_175848 | 240 bp | 5’-GCTGCTCACAGGGCCCGAT  ACTTC-3’ | 5’-CCTTTCGAGCTCAGTGCAC  CACAAAAC-3’ |
| **hGdf3** | NM_020634 | 667 bp | 5’-TTATGCTACGTAAAGGAGC  TGGG-3’ | 5’-TGCCAACCCAGGTCCCGGA  AGTT-3’ |
